# Extramedullary multiple myeloma patient-derived orthotopic xenograft with a highly altered genome: combined molecular and therapeutic studies

**DOI:** 10.1242/dmm.048223

**Published:** 2021-07-15

**Authors:** Lourdes Farre, Gabriela Sanz, Neus Ruiz-Xivillé, Manuel Castro de Moura, Juan Francisco Martin-Tejera, Samuel Gonçalves-Ribeiro, Maria Martinez-Iniesta, Monica Calaf, Jose Luis Mosquera, José Ignacio Martín-Subero, Isabel Granada, Manel Esteller, Eva Domingo-Domenech, Fina Climent, Alberto Villanueva, Anna Sureda

**Affiliations:** 1 Group of Chemoresistance and Predictive Factors, Subprogram Against Cancer Therapeutic Resistance, Catalan Institute of Oncology, Oncobell Program, Bellvitge Biomedical Research Institute (IDIBELL), 08908 L'Hospitalet del Llobregat, Barcelona, Spain; 2 Department of Clinical Hematology, Catalan Institute of Oncology, Bellvitge Biomedical Research Institute, 08908 L'Hospitalet del Llobregat Barcelona, Spain; 3 Hematological Laboratory, Germans Trias i Pujol Hospital, Catalan Institute of Oncology, 08916 Badalona, Barcelona, Spain; 4 Cancer and Leukemia Epigenetics and Biology and Experimental and Clinical Hematology Programs, Josep Carreras Leukaemia Research Institute, 08916 Badalona, Barcelona, Spain; 5 IDIBELL Bioinformatic Unit – Bellvitge Biomedical Research Institute (IDIBELL), 08908 L'Hospitalet del Llobregat, Barcelona, Spain; 6 Biomedical Epigenomics Group, Institut d'investigacions Biomèdiques August Pi I Sunyer (IDIBAPS), University of Barcelona, 08036 Barcelona, Spain; 7 Institució Catalana de Recerca i Estudis Avançats, 08010 Barcelona, Spain; 8 Centro de Investigación Biomédica en Red de Cancer, Carlos III Institute of Health, 28029 Madrid, Spain; 9 Physiological Sciences Department, School of Medicine and Health Sciences, University of Barcelona, 08036 Barcelona, Spain; 10 Department of Pathology, Hospital Universitari de Bellvitge – Bellvitge Biomedical Research Institute, 08907 L'Hospitalet de Llobregat, Barcelona, Spain; 11 Xenopat S.L., Business Bioincubator, Bellvitge Health Science Campus, 08907 L'Hospitalet de Llobregat, Barcelona, Spain

**Keywords:** Extramedullary multiple myeloma, Patient derived orthoxenograft, Genetic alterations, Epigenetic alterations, Therapeutic responses

## Abstract

Extramedullary multiple myeloma (EMM) has an overall survival of 6 months and occurs in 20% of multiple myeloma (MM) patients. Genetic and epigenetic mechanisms involved in EMM and the therapeutic role of new agents for MM are not well established. Besides, well-characterized preclinical models for EMM are not available. Herein, a patient-derived orthotopic xenograft (PDOX) was generated from a patient with an aggressive EMM to study in-depth genetic and epigenetic events, and drug responses related to extramedullary disease. A fresh punch of an extramedullary cutaneous lesion was orthotopically implanted in NOD.*Cg-Prkdc^scid^Il2rg^tm1Wjl^*/SzJ(NSG) mouse. The PDOX mimicked histologic and phenotypic features of the tumor of the patient. Cytogenetic studies revealed a hyperploid genome with multiple genetic poor-prognosis alterations. Copy number alterations (CNAs) were detected in all chromosomes. The *IGH* translocation t(14;16)(q32;q23)*IGH/MAF* was already observed at the medullary stage and a new one, t(10;14)(p?11-12;q32), was observed only with extramedullary disease and could be eventually related to EMM progression in this case. Exome sequencing showed 24 high impact single nucleotide variants and 180 indels. From the genes involved, only *TP53* was previously described as a driver in MM. A rather balanced proportion of hyper/hypomethylated sites different to previously reported widespread hypomethylation in MM was also observed. Treatment with lenalidomide, dexamethasone and carfilzomib showed a tumor weight reduction of 90% versus non-treated tumors, whereas treatment with the anti-CD38 antibody daratumumab showed a reduction of 46%. The generation of PDOX from a small EMM biopsy allowed us to investigate in depth the molecular events associated with extramedullary disease in combination with drug testing.

## Abstract (precis)

**Summary:** The generation of a patient-derived orthotopic xenograft allowed in-depth molecular and therapeutic studies in aggressive extramedullary multiple myeloma, revealing new cytogenetic aberrations, mutations, hypermethylated genome and drug responses.

## INTRODUCTION

Multiple myeloma (MM) accounts for 13% of hematological malignancies (Becker, 2011; Howlader et al., 2009). It is characterized by clonal proliferation of neoplastic plasma cells within the bone marrow (Bladé et al., 2011). Survival of patients with MM has increased significantly in the last two decades due to the use of high-dose therapy followed by autologous stem cell transplantation and the emergence of multiple novel therapies (Anderson et al., 2012). However, over time, MM patients often can exhibit worse and less durable responses to the different lines of treatment, and even develop extramedullary disease in 10-30% of cases (Bladé et al., 2011; Varga et al., 2015; Bhutani et al., 2020). In extramedullary multiple myeloma (EMM), myeloma cells become independent of bone marrow microenvironment, infiltrate other organs and/or circulate freely in the blood (Bladé et al., 2011). The most common sites for extramedullary disease were skin, soft tissues and liver (Usmani et al., 2012). The outlook for patients with EMM is poor, with survival no longer than 3 years (Bhutani et al., 2020; Bladé et al., 2011). EMM management is challenging due to the lack of a current rationale favoring a specific therapeutic class among those available (Touzeau and Moreau, 2016). The current treatment strategy is tailored to patient age and fitness. The molecular mechanisms involved in EMM are not well known. Furthermore, relevant *in vivo* preclinical models for EMM are not available. These difficult in-depth studies combined molecular and therapeutic approaches for extramedullary disease. Patient-derived orthotopic xenografts (PDOX), in which a human tumor biopsy is implanted in immunodeficient mice in the same organ as the tumor is grown in the patient, are the most advanced *in vivo* tumor preclinical models, as they reproduce better patient tumor behavior and therapeutic responses than other models, such as subcutaneous xenografts or models based on the injection of cell lines (Byrne et al., 2017). Herein, we aimed to generate a PDOX derived from an extramedullary cutaneous lesion of a resistant and rapidly progressive EMM patient with very poor outcomes to exhaustively investigate the genetic and epigenetic events and therapeutic responses related to extramedullary disease.

## RESULTS

### EMM patient and PDOX model generation

The PDOX was derived from an EMM cutaneous lesion of a 62-year-old female patient who was diagnosed with IgG kappa MM, stage IIIA (Durie–Salmon classification), in July 2016. During diagnosis, the patient's bone marrow aspirate demonstrated a 44% infiltration by plasma cells with CD38 positive and CD56 and CD119 negative expression. She rapidly progressed to EMM with the presence of multiple cutaneous lesions after the first line of treatment consisted of a combination of bortezomib, thalidomide and dexamethasone (VTD). At extramedullary disease, multiple cutaneous lesions were positive for dermal infiltration by plasma cell neoplasm (Kappa positive, CD138 and MUM-1 positive; Ki67 positivity of 90%). Massive bone marrow infiltration by plasmoblasts, progressive left pleural effusion that was also specific and pleural thickening and thoracic lymphadenopathies were also observed. The patient died five months later after showing no response to two other lines of treatment that consisted of the salvage chemotherapy regime D-PACE (dexamethasone, cisplatin, Adriamycin, cyclophosphamide and etoposide) in November 2016, and the combination of methotrexate and cytarabine, which started in December 2016. Details of the patient clinical course and treatment schemes are described in Materials and Methods.

**Fig. 1.**
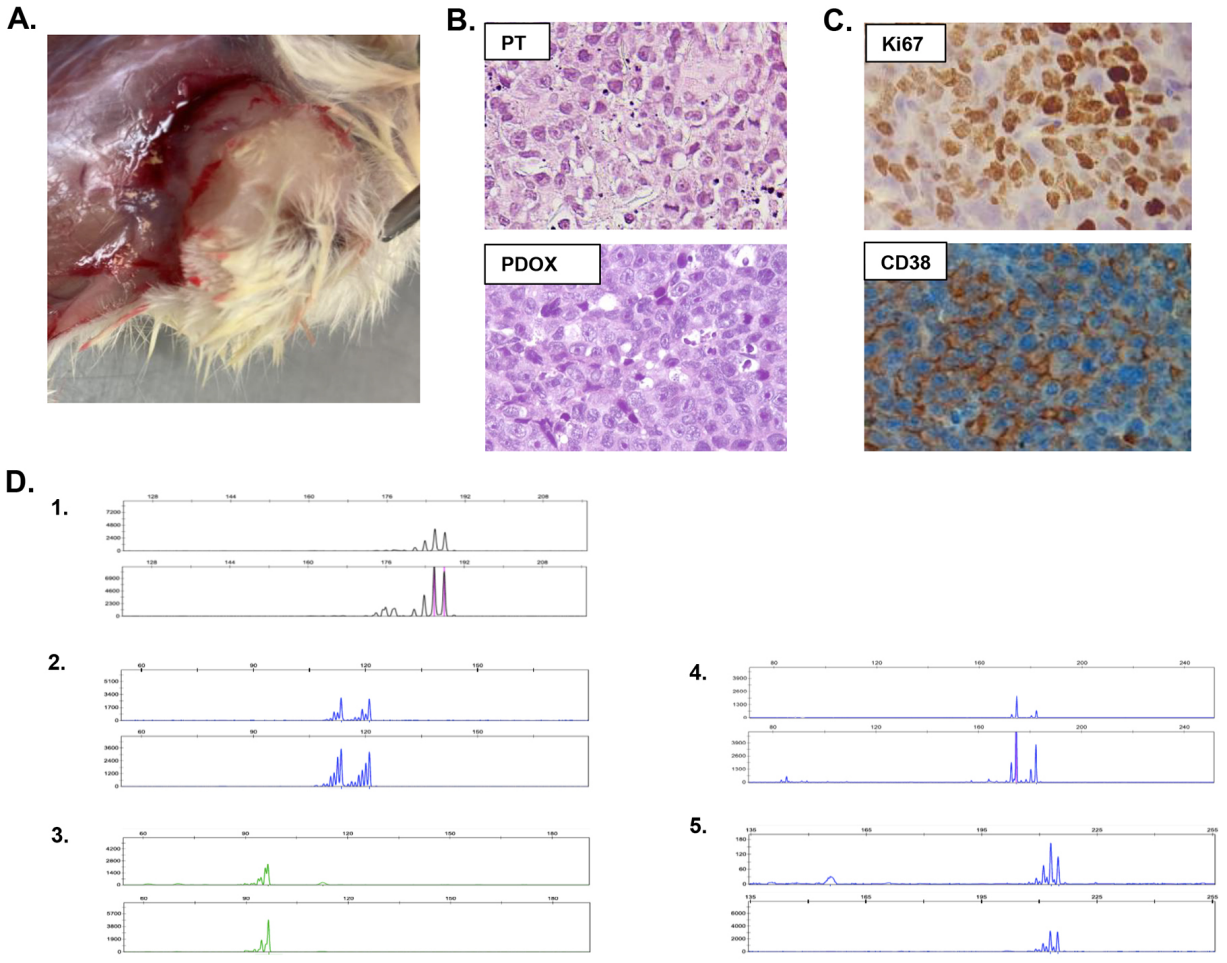
**Characterization of EMM PDOX.** (A) Macroscopic appearance of PDOX. (B) H&E staining of the patient's extramedullary lesion (PT, upper panel) and xenografted tumor (PDOX, lower panel), showing histological similarities between the original tumor sample and the originated EMM PDOX (at 400× magnification). (C) EMM PDOX cells showed a Ki67 positivity of 90% and CD38 expression, as observed in the patient's precursor tumor. (D) Paired microsatellite genotyping of PDOX and the patient's lesion from which it was derived. The genetic match between the generated PDOX and the patient's tumor was assessed by microsatellite genotyping. The microsatellites used for tumor genotyping were (1) D5S299, (2) D5S346, (3) D3S1612, (4) D5S82 and (5) D3S3564. For each microsatellite, the upper panel corresponds to the patient's biopsy and the lower panel corresponds to PDOX.

A fresh small punch biopsy of an extramedullary skin lesion was obtained at the beginning of the third line of treatment to generate the EMM-derived PDOX model (EMM PDOX). The model was successfully generated by orthotopic cutaneous surgical implantation in one NOD.*Cg-Prkdc^scid^Il2rg^tm1Wjl^*/SzJ (NSG) mouse. The 1 mm^3^ tumor fragment implanted in the mouse reached a volume of 1 cm^3^ 30 days after implantation, showing a rapid growth rate. The macroscopic aspect of the xenograft tumor is shown in Fig. 1A. The mouse was sacrificed, and the tumor was re-implanted into two other animals to expand and to perpetuate the model, and then processed in order to exhaustively investigate this aggressive EMM case by way of histologic, phenotypic, genetic and epigenetic studies. EMM PDOX histology was similar to its biopsy precursor, showing groups of large cells with poorly differentiated morphology, broad cytoplasm and nuclei with prominent nucleoli and multiple mitotic figures (Fig. 1B). EMM PDOX cells showed a Ki67 positivity of 90% and were positive for CD38 expression, as observed in EMM patient lesions (Fig. 1C). The genetic matching between EMM PDOX and the patient's biopsy was also investigated, and was demonstrated by microsatellite genotyping using six different microsatellite markers as shown in Fig. 1D.

**Fig. 2.**
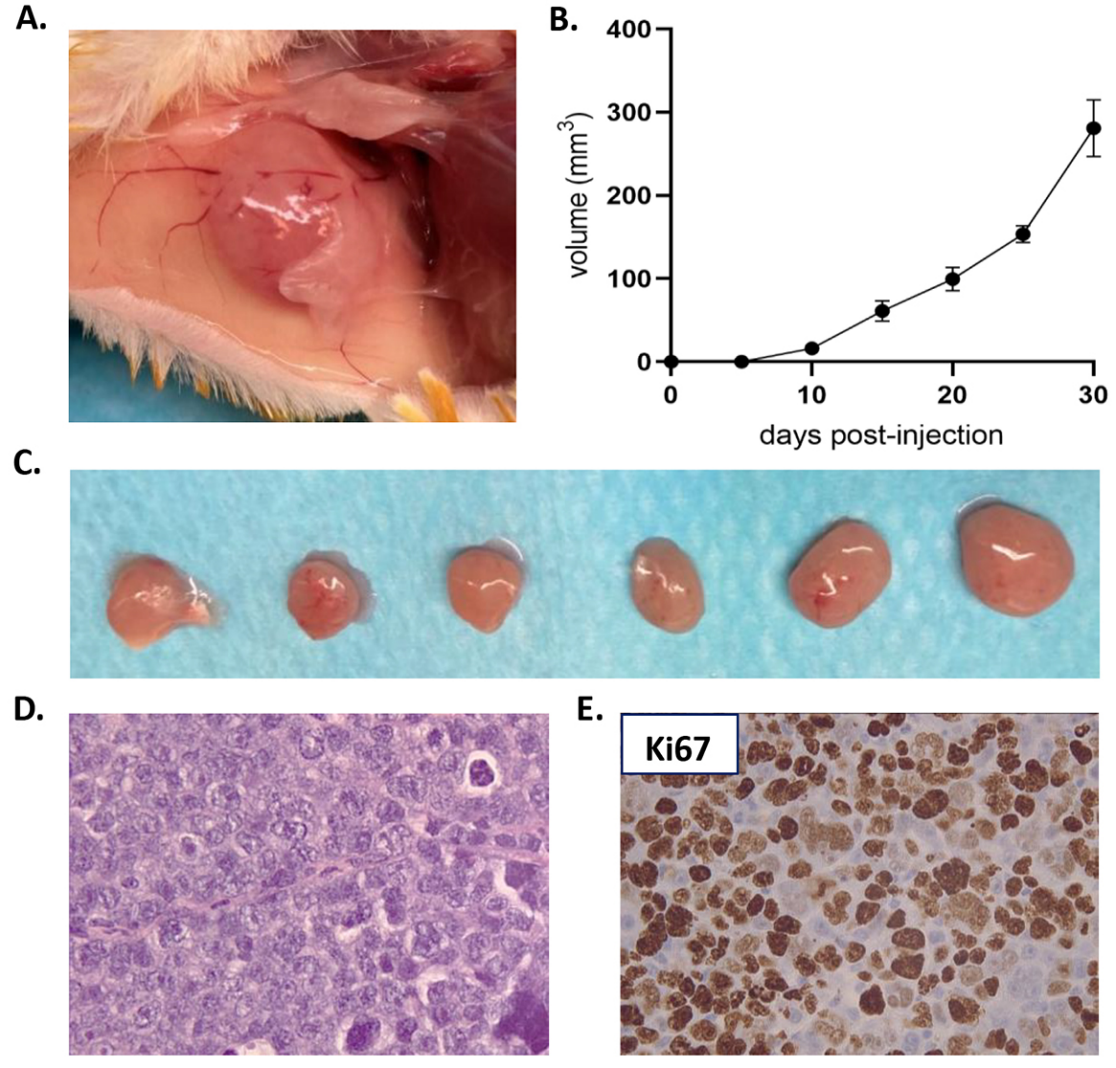
**Derived EMM cell line generated plasmocytomas when re-implanted in NSG mice.** (A) Representative image of a plasmocytoma derived from EMM cell line by intradermal injection of 1×10^6^ cells soaked in Matrigel in the flank of an NSG mouse. (B) Tumor growth rate of plasmocytomas generated after the intradermic injection of 1×10^6^ cells of the EMM cell line (*n*=6). (C) Tumors reached a mean volume of 280±34 mm^3^ at 30 days post-injection. (D) H&E staining of the cell line-derived plasmocytomas showing histological similarities with the PDOX and the patient's biopsy (at 400× magnification). (E) Cell line-derived plasmocytomas showed a high Ki67 expression as observed in the EMM PDOX (at 400× magnification). Data are mean±s.e.m.

An EMM cell line was also derived from tumor xenograft, and was grown *in vitro* in a suspension culture. Most of the cells formed cell clumps and showed a doubling time of 41±6 h (mean±s.d.). The EMM cell line was maintained until passage 10, and after each passage an aliquot was cryopreserved. During each passage, clumps were brought into a single-cell suspension and the cells spontaneously formed clumps again. The EMM cell line also grew and generated new plasmocytomas when injected in NSG mice. A total of 1×10^6^ cells soaked in Matrigel were intradermally injected in two back flanks of NSG mice (*n*=6 injections in three NSG mice), generating tumor masses in all cases (Fig. 2A). Plasmocytomas reached a mean volume of 280±34 mm^3^ (mean±s.e.m.) 30 days post-injection (Fig. 2B,C). Cell line-derived plasmocytomas showed a similar histology to the original PDOX tumor and the patient's tumor, as well as similar Ki67 expression (Fig. 2D,E).

### Genetic and epigenetic analysis of EMM PDOX

**Fig. 3.**
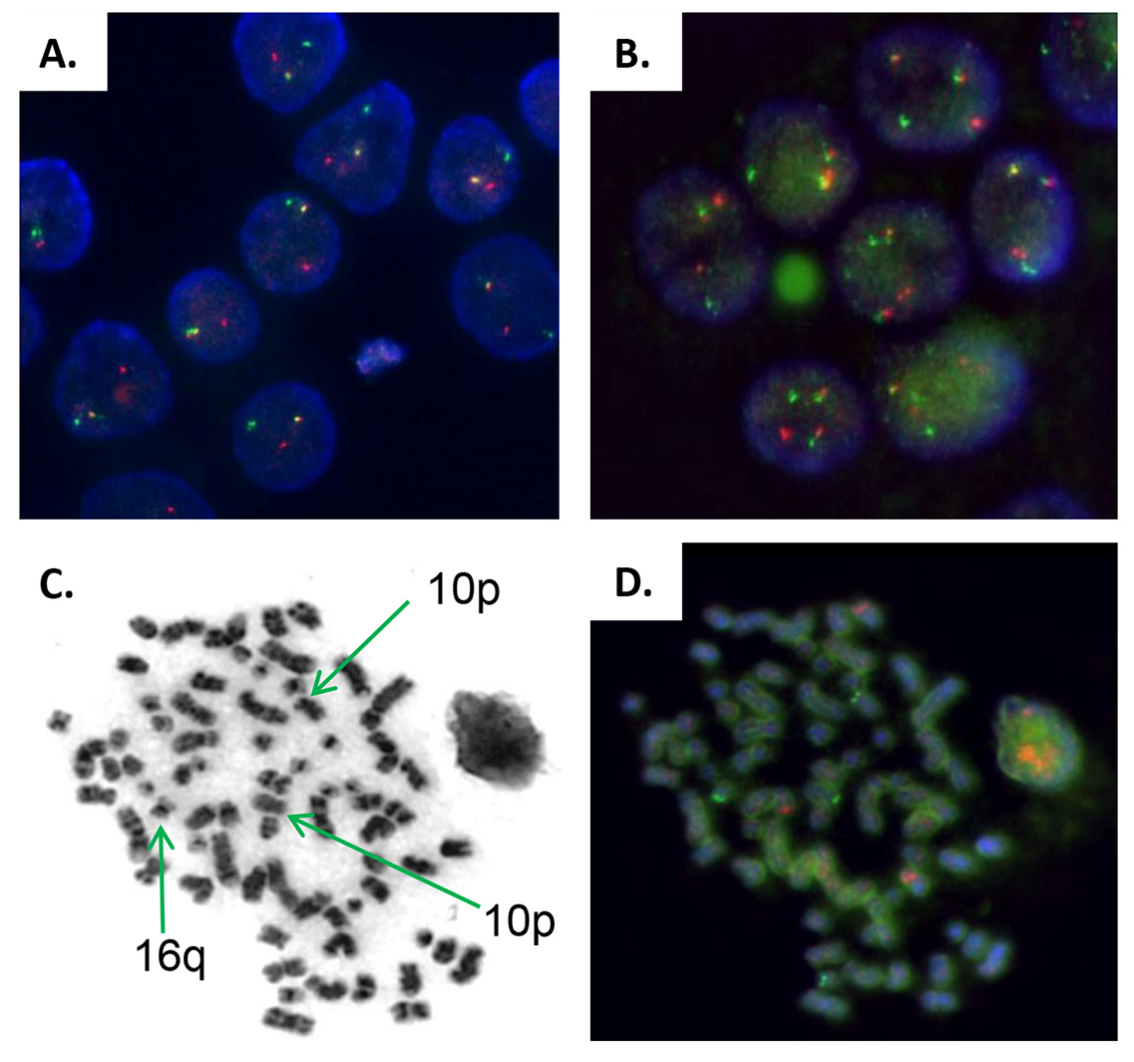
**Fluorescence *in situ* hybridization analysis of the immunoglobulin heavy chain gene locus (*IGH*).** (A) *IGH* rearrangement was observed in only one chromosome at diagnosis before the occurrence of extramedullary disease (Break Apart Probe). (B) FISH analysis of t(14;16) *IGH-MAF* rearrangement of EMM PDOX showing the translocation in the two chromosomes. (C,D) Micrograph of G-band metaphase chromosomes of the PDOX (C) and FISH (D) analysis of *IGH* rearrangements, identifying chromosomes 10 (region p) and 16 (region q) implicated in IGH t(14;16)(q32;q23) and t(10;14)(p?11-12;q32) translocations. The later translocation was not detected at MM diagnosis. All images are at 1000× magnification.

Karyotype analysis of EMM PDOX identified a hyperploid genome. Fluorescence *in situ* hybridization (FISH) analysis for *IGH* rearrangements was performed in the EMM PDOX and additionally in the remaining patient's bone marrow aspirate obtained at MM diagnosis, already at medullary stage (Fig. 3A,B). Both samples showed positivity for the t(14;16)(q32;q23)*IGH/MAF* rearrangement but were negative for the t(4;14)(p16;q32)*IGH/FGFR3* rearrangement. The EMM PDOX also showed the t(10;14)(p?11-12;q32) IGH translocation, identified at G banding resolution, that was not observed in the bone marrow plasma cells at medullar stage (Fig. 3C,D).

**Fig. 4.**
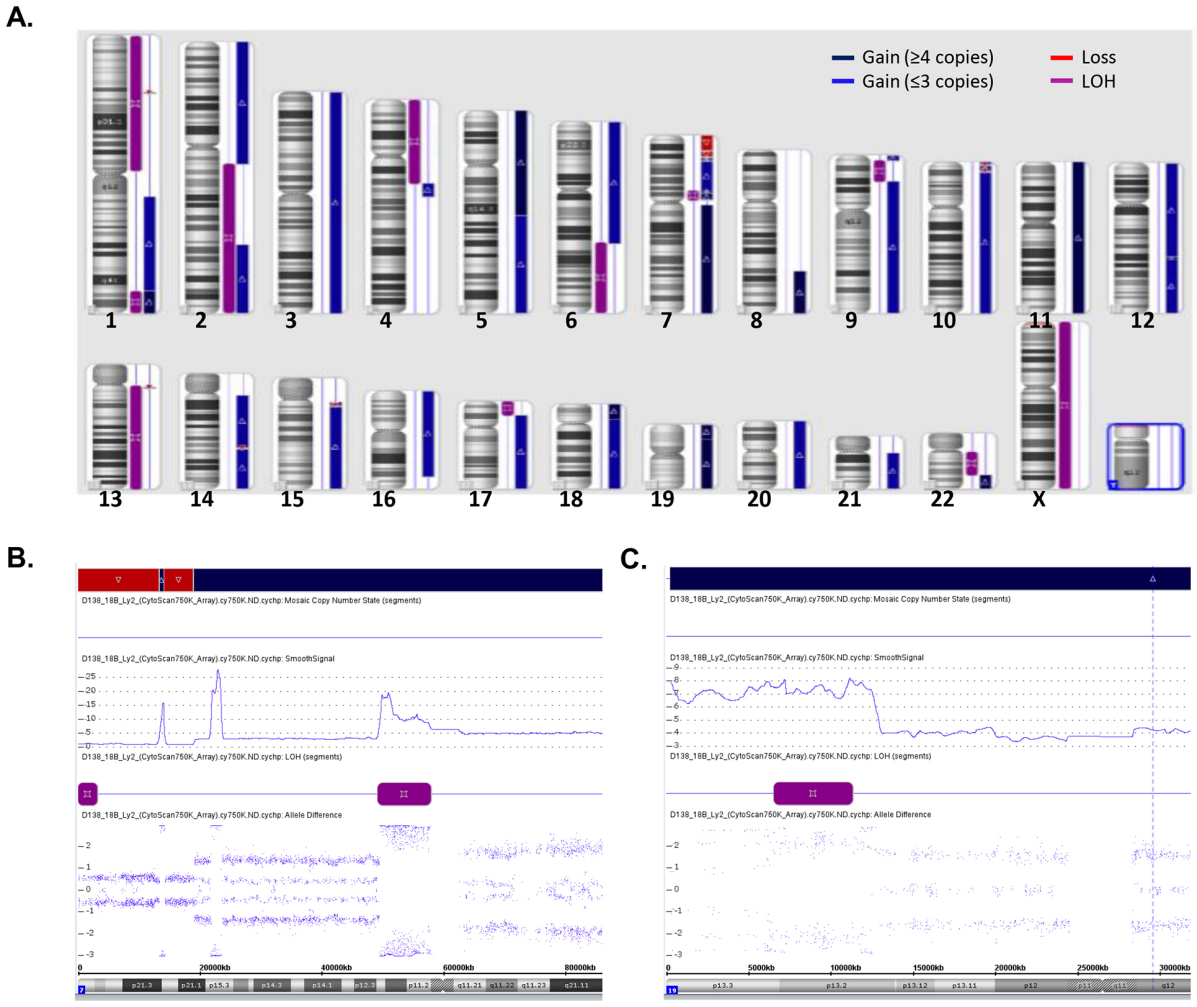
**Cytogenetic abnormalities observed in EMM PDOX.** (A) Diagram representing the cytogenetic abnormalities observed in EMM PDOX by single-nucleotide polymorphism genotyping microarray performed using a CytoScan 750 K Array. CNAs are extensively distributed along all chromosomes and included gains, losses and LOH. (B,C) Presence of extensive intrachromosomal CNAs. Details of CNA analysis of the short arm of chromosome 7 (B) showing a highly disturbed genome with gains of up to 28 copies concomitant with extensive genetic losses and LOH and detail of CNA in the short arm of chromosome 19 (C) showing a gain region of 12.8 mb with seven copies.

Genetic imbalance analysis with a Cytoscan 750K array in the EMM PDOX showed a highly disturbed genome with multiple CNAs in all chromosomes, including gains, losses and/or loss of heterogeneity (LOH) (Fig. 4A). Trisomy of chromosomes 3, 5, 7, 9, 15, 17 and 21, and tetrasomy of 11 and 19 were observed. The most frequent CNAs were extensively intrachromosomic amplifications. In chromosomes 1, 5, 7, 9, 11, 12 and 19, gains of four or more copies were detected. Among them, chromosome 7 was notable with 10 to 18 copies in the 7p12 region, 20 to 28 copies in the 7p15 region and 16 copies in the 7p21 region, as well as chromosome 19 with 7 copies in 19p13 (Fig. 4B,C; Table S1). CNAs affected an exceptionally large number of genes that are annotated in Table S1. Among others, we underlined the following alterations previously associated with poor prognosis: losses in 1p32.3 affecting the *FAF1* and *CDKN2C* genes; gains in 1q21 affecting the *CKS1B* gene; LOH of chromosome 13; and LOH in the short arm of chromosome 17 (13.3 mb), which includes the *TP53* gene. In chromosome 7 (p15.3), a region of 1210.71 kb, with a gain of 28 copies, affected 17 genes, including the gene encoding the cytokine IL6.

**Fig. 5.**
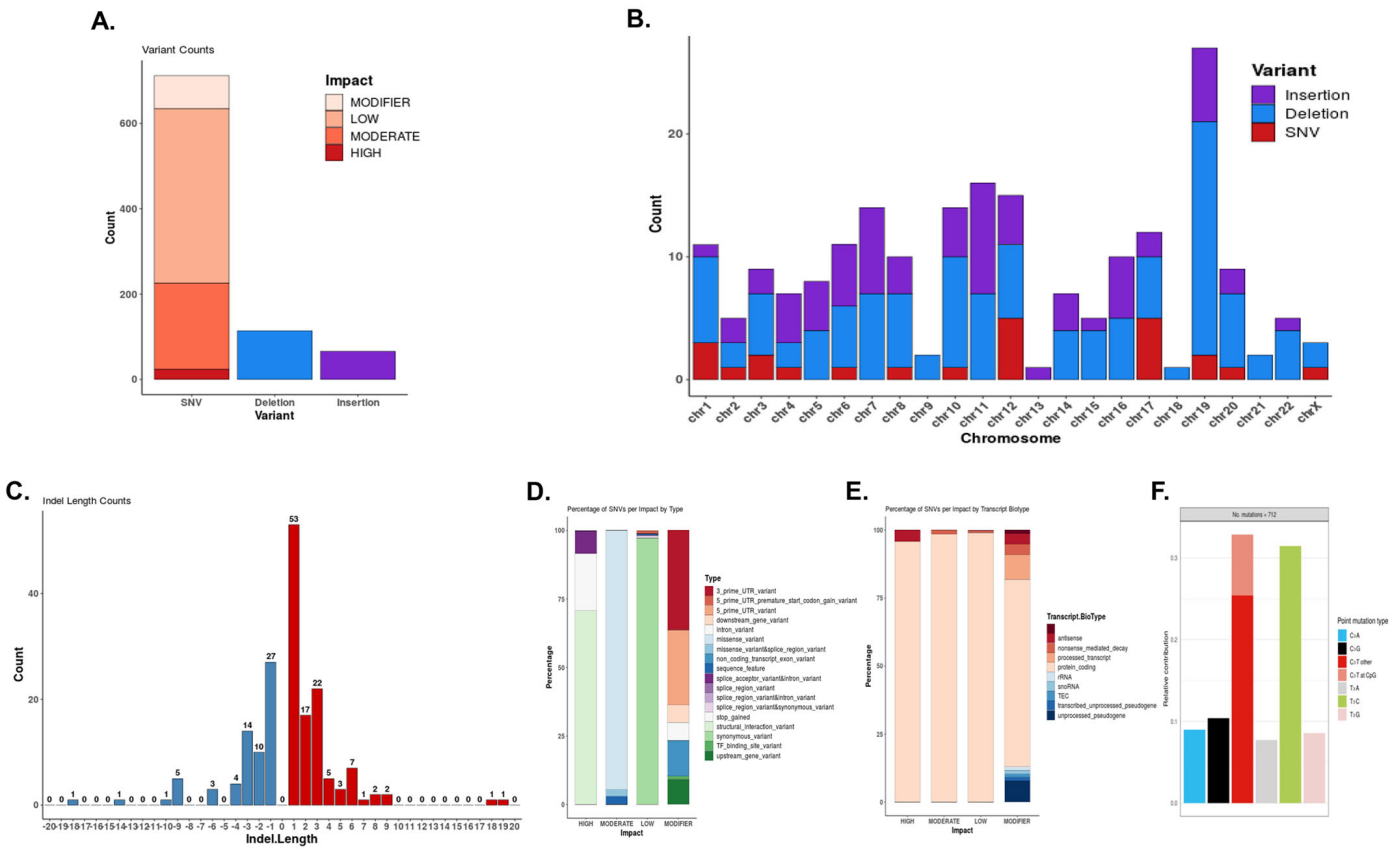
**Variants in EMM PDOX detected by whole-exome sequencing.** (A) Total number of SNVs classified as high, moderate and low impact and modifier, and insertions and deletions. (B) Variant counts per chromosome in terms of high-impact SNVs, insertions and deletions (indels). (C) Counts of indels by length. In the analysis, indels were considered as deletions or insertions of up to 20 bp. (D,E) Percentage of SNVs per impact by type (D) and by transcript biotype (E). All 712 SNVs were included in the analysis. (F) Relative concentrations of point mutation types detected. To obtain the COSMIC signatures contribution, it was assumed that all SNVs detected corresponded to somatic mutations.

Besides cytogenetic abnormalities, 712 single nucleotide variations (SNVs) (24 of high, 202 of moderate, 409 of low impact and 77 modifier), 114 deletions and 66 insertions were found by whole-exome sequencing in the EMM PDOX (Fig. 5A). These alterations were distributed in all chromosomes, with a high number of variants in chromosome 19 but fewer in chromosomes 9, 13, 18 and 21 (Fig. 5B). In our analysis, indel length was limited to 20 bp, with the most frequent being those with 1 to 3 nucleotides for both insertions and deletions (Fig. 5C). Genes affected by high impact SNVs are annotated in Table S2. Among them, we highlight genes implicated in cell cycle regulation (*TP53*, *cdc7*, *CDC25A*, *TATD1* and *PDE5*), gene transcription (*HDAC4*), cell-to-cell adhesion (*CTNNA3*) and metabolic processes (*AMD1* and *GAPDH*). Additional analysis by type/transcript biotype percentages and transversion/transition distributions are shown in Fig. 5D-F.

**Fig. 6.**
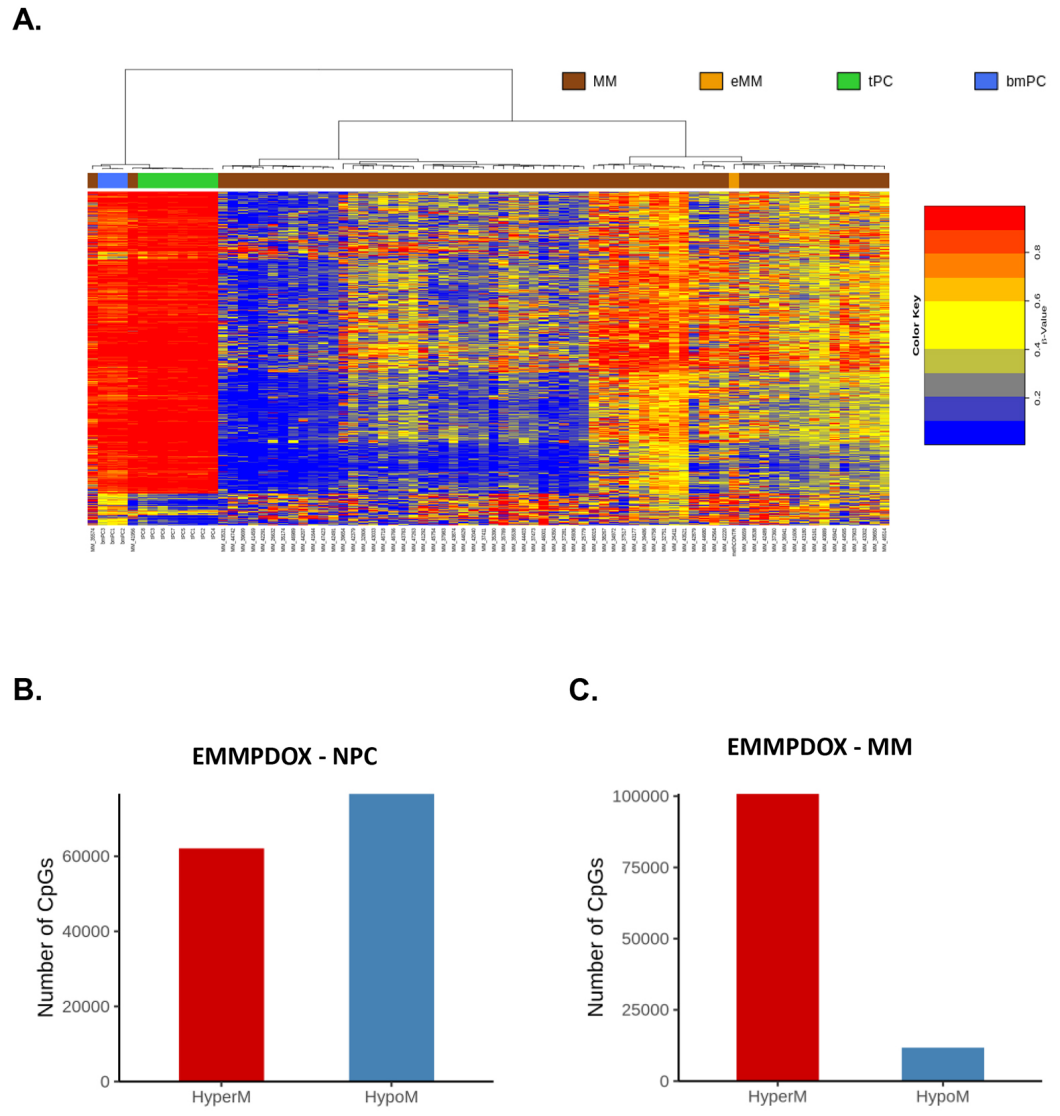
**Whole-genome methylation analysis of EMM PDOX.** (A) Unsupervised hierarchical clustering analysis of EMM PDOX, MM, NPCs from tonsil (tPC) or NPCs from bone marrow (bmPC). The 5000 CpG sites with the most variable methylation values were used. (B) Differentially methylated CpGs in EMM PDOX compared to NPCs and (C) newly diagnosed and chemotherapy-naïve MM patients (Agirre et al., 2015). Compared with NPCs, we identified 138,645 differentially methylated CpGs, including 55.3% hypomethylated and 44.7% hypermethylated. As compared with MM, we identified 112,551 differentially methylated CpGs, including 10.4% hypomethylated and 89.6% hypermethylated. Epigenetic data were obtained using an Infinium MethylationEPIC BeadChip array (Illumina) for EMM PDOX (from four different fragments of the tumor) and using a 450 K methylation array (Illumina) for MM and NPCs (Agirre et al., 2015). To compare the results with the published 450 K methylation arrays, only the common probes with EPIC were taken into account, and average methylation values were used for each group.

Whole DNA methylome of the EMM PDOX was also evaluated and compared with a published dataset of 101 newly diagnosed and chemotherapy-naïve MM patients and normal plasma cells (NPCs) (Agirre et al., 2015) (Fig. 6A). As compared to NPCs, we identified a total of 138,648 differentially methylated CpGs in the EMM PDOX, including 55.3% hypomethylated and 44.7% hypermethylated (Fig. 6B). We then compared the methylome of the EMM PDOX with the methylomes of MM patients and identified 112,551 differentially methylated CpGs in the EMM PDOX, including 10.4% hypomethylated and 89.6% hypermethylated, thus observing that EMM PDOX was more hypermethylated than the MM cases (Fig. 6C). Methylation-regulated genes in EMM PDOX in comparison to MM cases are annotated in Table S3. Distributions of hyper/hypomethylated sites by genome functional regions are shown in Fig. S1. Even a significant number of CpGs that were differentially methylated between EMM PDOX and NPCs, the percentage of total hypermethylated and hypomethylated CpGs, and their distribution by genome functional regions, were quite similar. The differentially methylated CpGs between EMM PDOX and MM cases were distributed more frequently in body regions for both hypermethylated and hypomethylated CpGs.

### Therapy responses in EMM PDOX

**Fig. 7.**
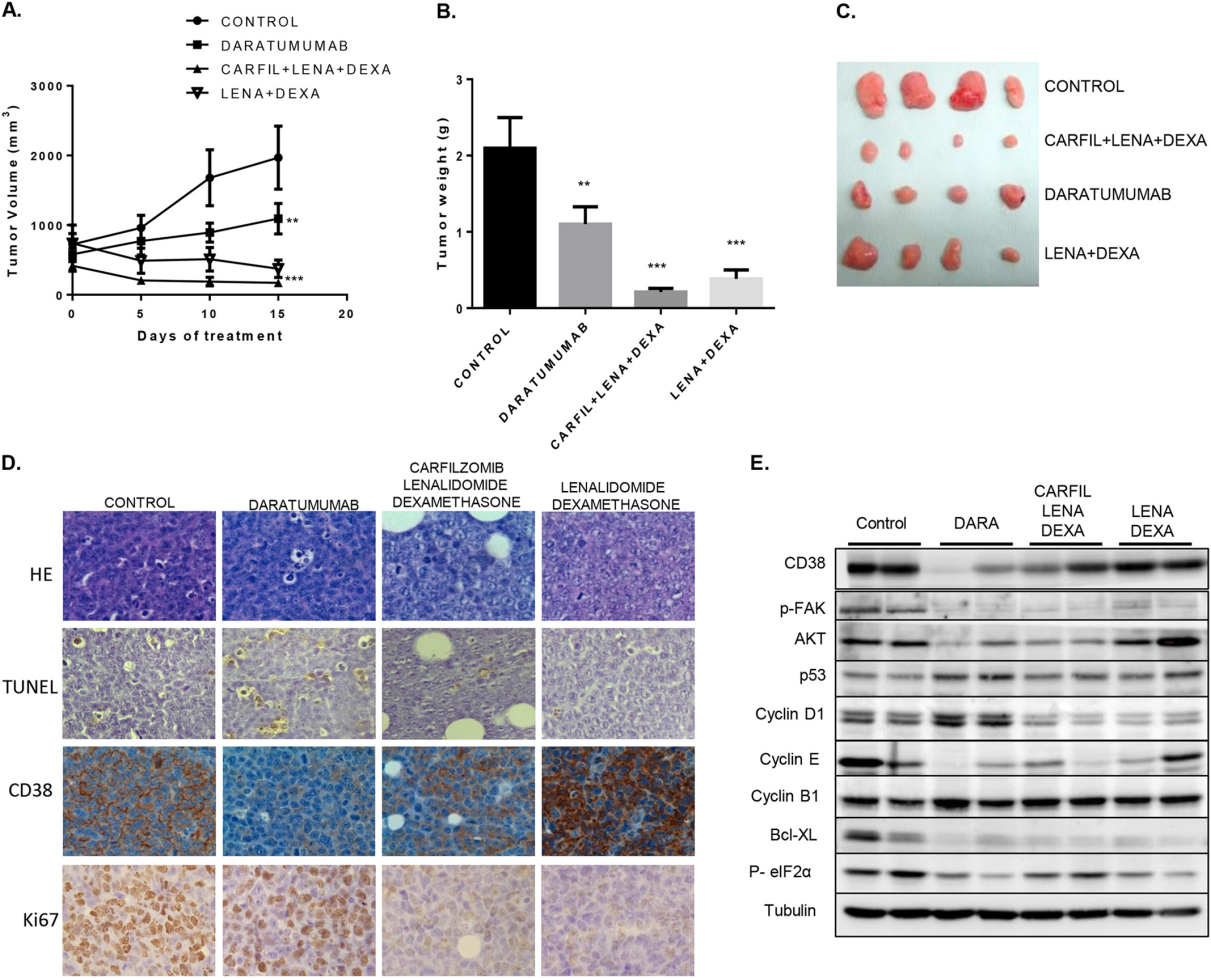
***In vivo* therapeutic assays using EMM PDOX.** EMM xenografts were expanded orthotopically to 40 additional NSG mice. After evidence of homogeneous tumor growth, animals were randomly distributed in four groups (*n*=10) and treated with saline (control), with daratumumab (DARA) in monotherapy, with the combination of lenalidomide (LENA) and dexamethasone (DEXA), or the combination of lenalidomide, dexamethasone and carfilzomib (CARFIL) for 15 days. No signs of animal toxicity were observed with any of these treatments. (A) Evaluation of tumor volumes during tumor treatment. (B) Tumor weights at the end of the treatment at mice sacrifice for the different experimental groups. (C) Representative diagram of tumors dissected at sacrifice for the different experimental groups. (D) H&E staining, IHQ evaluation of Ki67 and CD38, and TUNEL assay (at 400× magnification). A decrease of CD38 expression was observed only in daratumumab-treated tumors. A decrease of Ki67 was observed after the treatment with lenalidomide-dexamethasone with or without carfilzomib, without a significant increase of apoptotic cells. A pro-apoptotic effect was observed in daratumumab tumors with a significant increase of apoptotic cells evaluated by TUNEL assay with respect to the control group without significant Ki67 expression change. (E) Western blot analysis of CD38 and the relevant proteins implicated in cell cycle control and apoptosis. A decrease of CD38 expression was observed also by western blot only in daratumumab-treated tumors. Data are mean±s.e.m.

Owing to the lack of response to high-dose chemotherapy regimens observed in the patient, we took advantage of the generated EMM PDOX to test new available therapies for MM not used with the patient from whom the PDOX derived, and which have not been explored much for extramedullary disease control. Tumor was expanded by orthotopical implantation in 40 additional NSG mice. We included the anti-CD38 antibody daratumumab in monotherapy (in this case EMM cells showed CD38 expression) and the combination of lenalidomide and dexamethasone with or without the proteosome inhibitor carfilzomib, as these schemes are recommended for the treatment of MM in patients who have received at least one previous therapy. A significant reduction in both tumor volume and weight was observed in all treated groups compared with control group (Fig. 7A-C). The highest antitumoral effect was achieved with the combination of lenalidomide, dexamethasome and carfilzomib, with a tumor weight reduction of 90% compared with tumors treated with vehicle (control tumors, *P*=0.0015), followed by the combination of lenalidomide and dexamethasone, which resulted in an 81% tumor weight reduction compared with control tumors (*P*=0.0030). Daratumumab had a significant but only partial response, showing a 46% tumor weight reduction compared with control tumors (*P*=0.043). No signs of animal toxicity were observed with any of these combinations. In the residual masses, we evaluated: (1) histological changes by Hematoxylin and Eosin (H&E) staining; (2) cellular proliferation by Ki67 staining (IHQ); (3) the presence of apoptotic cells by TUNEL assay; (4) the expression of CD38 by immunohistochemistry and (5) the molecular regulation of cell cycle, cell death and endoplasmic reticulum stress pathways by western blotting.

In lenalidomide plus dexamethasone-treated tumors and in those that additionally received carfilzomib, TUNEL assay showed a similar number of apoptotic cells compared with control tumors, and a decrease of Ki67 expression was observed (Fig. 7D). Western blot analysis showed a decrease of cyclin D1, cyclin E and Bcl-X_L_ expression, and FAK phosphorylation. In lenalidomide and dexamethasone-treated tumors, a decrease of eIF2α phosphorylation in comparison to control tumors was detected, which was not observed in tumors also treated with carfilzomib. The addition of carfilzomib to the lenalidomide and dexamethasone treatment was associated with a decrease in AKT expression (Fig. 7E).

The specificity and effectiveness of daratumumab treatment were confirmed by a decrease of CD38 expression detected by IHQ and western blot in residual tumor masses, observed only for this treatment (Fig. 7D,E). Daratumumab response was associated with an increase of apoptosis (evaluated by TUNEL assay) compared to control tumors but similar Ki67 expression (Fig. 7D). In Daratumumab-treated tumors, an increase of p53 and cyclin D1 and B1 expression, and a decrease of AKT, cyclin E and Bcl-X_L_ expression and FAK and eIF2α phosphorylation were observed (Fig. 7E).

## DISCUSSION

The generation of an EMM PDOX starting from a small fresh punch of an extramedullary cutaneous lesion allowed us to investigate in depth the molecular alterations in combination with the therapeutic responses of an aggressive case of MM that progressed rapidly to extramedullary disease. PDOX have been demonstrated to be a powerful tool for expanding patient tumor tissue while maintaining their histological and genetic features, supporting the detection of new genetic and epigenetic events associated with extramedullary disease and allowing parallel and rational evaluation of *in vivo* drug response.

The molecular mechanisms involved in EMM are not well known. In the case shown here, a highly disturbed genome with IGH translocations, wide chromosomal imbalances, SNVs and a different methylation pattern to MM cases were detected. Chromosomal translocations involving IGH genes have a central role in MM, and their incidence increases with each successive stage of disease (Bergsagel and Kuehl, 2018; Walker et al., 2018). This aggressive case already presented the t(14;16)(q32;q23)IGH/MAF translocation in bone marrow tumoral plasma cells at diagnosis, which, mainly in combination with negative expression of CD56, as observed here, has been associated with an unfavorable outcome in MM (Bhutani et al., 2020; Narita et al., 2015; Shah et al., 2018; Walker et al., 2018). Furthermore, extramedullary tumor in the PDOX additionally presented the t(10;14) (p?11-12;q32) translocation that could eventually be related to disease progression to extramedullary disease in this case. To our knowledge, this translocation has not been previously reported in MM (Bhutani et al., 2020; Shah et al., 2018; Walker et al., 2018) but has been in six cases of chronic lymphocytic leukemia and in one case of nodal marginal zone B-cell lymphoma (Martín-Subero et al., 2007; Rouhigharabaei et al., 2013).

Even trisomy in odd chromosomes is commonly associated with a good prognosis in MM. This case also presented a lot of previously reported poor prognostic cytogenetic abnormalities that could contribute to the observed poor outcome, such as the loss in 1p32.3 and gain in 1q21 (An et al., 2014; Avet-Loiseau et al., 2009; Besse et al., 2016; Boyd et al., 2011; Hebraud et al., 2015; Shah et al., 2018; Walker et al., 2010). The LOH observed in the short arm of chromosome 17(13.3 mb) was indicative of a deletion of this region that includes the *TP53* gene. This poor prognosis alteration was not detected in the patient at the MM diagnosis and may also be related to extramedullary disease in this case. Monosomy of chromosome 13 and deletion 1p13.2-34.2 have been reported in another multidrug-resistant EMM patient (Egan et al., 2013), suggesting that these events may have a role or be frequent in extramedullary disease. In some chromosomes, gains of more than five copies were detected. A region of 1210.71 kb in 7p15 with a gain of 28 copies contained the gene encoding for the IL6 cytokine that has been reported as a crucial factor for the proliferation and survival of myeloma cells (Rosean et al., 2014).

Among genes with high-impact point mutations observed in this case, only *TP53* was described as a driver in MM (Walker et al., 2018). Mutations in the other genes have not been previously reported in MM or EMM (Bhutani et al., 2020; Morgan et al., 2012; Rasche et al., 2017; Walker et al., 2018) but some of them have been implicated in MM pathogenesis. For instance, *HDAC4* is implicated in MM cell survival and migration, and panobinostat, a pan-HDAC inhibitor, in combination with a proteasome inhibitor and dexamethasone, has improved survival in relapsing and refractory MM patients (Imai et al., 2019). An inhibitor of *PDE5*, tadalafil, has been demonstrated to have a dramatic and durable anti-myeloma immune and clinical response in a patient with end-stage relapsed/refractory MM (Noonan et al., 2014). An inhibitor of Cdc7, the PHA-767,491, has been reported to block proliferation and to induce apoptosis of MM cell lines (Coyne et al., 2009). It has also been described that *STK4* has a role in MM cell survival, and STK4 inhibitors may represent novel therapeutic options for patients with MM (Cottini et al., 2014).

Regarding methylation, the rather balanced proportion of hyper/hypomethylated sites observed in this EMM case contrasts with the previously reported widespread hypomethylation in newly diagnosed and chemotherapy-naïve MM patients (Agirre et al., 2015). Indeed, the ratio of hyper/hypomethylated sites in this EMM case was more similar to NPCs than to MM, although methylation in this EMM case and in NPCs affected different CpGs. A higher degree of hypermethylation has also been observed in plasma cell leukemia in comparison to MM, and has been related to independence from the bone marrow microenvironment (Walker et al., 2011). It could be interesting to further investigate whether DNA hypermethylation plays a role in the progression of MM to more aggressive forms, in contrast with the hypomethylation process observed in the transition from NPC to MM. Differently hypermethylated CpGs between this EMM case and MM cases were preferably distributed within gene bodies and intergenic regions.

The role of new agents in the treatment success of EMM is still poorly understood. Several clinical reports demonstrate effectiveness of bortezomib (Patriarca et al., 2005; Rosiñol et al., 2006), as well of lenalidomide and pomalidomide (Ito et al., 2013; Nakazato et al., 2012). The data in EMM with incorporation of carfilzomib are not readily available. There are limited data regarding the efficacy of daratumumab, either alone or in combination, in EMM, although information coming from retrospective analyses does not seem to be very optimistic, with modest (Jullien et al., 2019) or low (Pick et al., 2018) responses to daratumumab. Moreover, expression levels of CD38 do not seem to be a predictive factor of response, as suggested by the responses found in our murine model. Our preclinical results may suggest the potential value of the combined therapies in EMM even with a highly disturbed genome.

Relevant and well-characterized preclinical models that simulate the patient's disease are critical tools for advancing understanding of the pathogenesis of EMM and in the preclinical discovery of new therapy strategies. Available preclinical models of MM mainly include myeloma models in immunocompetent mice (Radl et al., 1988; Fowler et al., 2009), xenograft models in severe combined immunodeficiency (SCID) and non-obese diabetic (NOD)/SCID mice, in which human MM cell lines or cells derived from MM patients are injected (Chauhan et al., 2006; Neri et al., 2008), and genetically modified models (Lwin et al., 2016). However, preclinical models for EMM are scarce and unique. PDOX models have been shown to mimic the same pattern of pharmacological response as tumor in the patient (Byrne et al., 2017; Hidalgo et al., 2014; Piulats et al., 2018; Capellá et al., 1999; Vidal et al., 2012), aspects that do not generally occur in xenograft models based on subcutaneous implantation whereby the tumor loses the tumor microenvironment, or based on the injection of cell lines that lose the heterogeneity of the tumor. The generation of EMM PDOX reported here also facilitated the creation of a novel EMM cell line capable of forming new plasmacytomas when injected in mice that will allow future complemental functional studies. As is the case with *in vivo* preclinical models, cell lines for EMM are scarce. Additionally, the approach presented here could also potentially support a personalized treatment strategy for EMM in a clinical setting.

## MATERIALS AND METHODS

### Patient data

The patient from whom the PDOX was derived was a 62-year-old female, with a past medical history of toxic nodular goiter and bilateral breast cancer in 2003, who was diagnosed with IgG kappa MM, stage IIIA (Durie–Salmon classification, International Score System of 2) in July 2016. The patient was started on VTD [1.3 mg/m^2^ bortezomib (days 1, 4, 8 and 11)], thalidomide up to 150 mg daily and 40 mg dexamethasone (days 1-4 and 9-12) for 4 cycles, with the objective of consolidating the response with autologous stem cell transplantation. After disease progression, she was started on D-PACE [40 mg dexamethasone, 10 mg/m^2^ cisplatin, 10 mg/m^2^ adriamycin, 400 mg/m^2^ cyclophosphamide and 40 mg/m^2^ etoposide (days 1-3), ×2 cycles] in November 2016, after disease progression with the reappearance of disseminated skin lesions. The patient began a third line consisting of 1000 mg/m^2^ methotrexate (day 1) and 1000 mg/m^2^ (days 2-3) in December 2016. Unfortunately, the clinical condition of the patient rapidly deteriorated and she died. The patient gave written consent to participate in the study. The Ethics Committee of the Bellvitge Hospital approved the study protocol, and the animal experimental design was approved by the IDIBELL animal facility committee. This study was conducted according to the principles expressed in the Declaration of Helsinki.

### PDOX generation

The PDOX model was generated by orthotopic cutaneous surgical implantation of the fresh small punch biopsy of an infiltrative extramedullary skin lesion in one 6-week-old female NOD.*Cg-Prkdc^scid^Il2rg^tm1Wjl^*/SzJ (NSG) mouse. Briefly, a cutaneous back shave biopsy of normal mouse skin was performed by removing a rhomboid area of ∼6 mm^2^ (4×3 mm), and then the small fragment of tumor tissue was anchored with a prolene 7.0 suture to the edge of the skin, fixing the tumor at four anchored sites coinciding with each corner. To improve the implantation process, the anchored skin tumor was protected by making a skin fold that was fixed with three points with 5.0 silk sutures (Vizoso et al., 2015). Tumor growth and animal weight were monitored once a week. The animal was sacrificed when tumor volume reached 1 cm^3^. Upon tumor growth, three fragments of ∼20 mm^3^ volume were re-implanted orthotopically in three other NSG mice in order to expand the tumor. Two fragments of similar volume were cryopreserved, and the remaining tumor mass was fragmented and processed for in-depth histologic, immunophenotypic, genetic and epigenetic studies.

### Histologic and immunohistochemistry analyses

For histologic analyses, a tumor fragment was fixed in formalin for 24 h, embedded in paraffin and stained with H&E. The histology of tumor PDOX was compared with the histology of extramedullary cutaneous lesion from which the PDOX was derived by a haematopathologist. For IHQ analysis, 3-µm slices of paraffin-embedded tissues were used. Primary antibodies used were monoclonal antibodies for Ki67 (RM-9106-51, Sigma-Aldrich) and CD38 (NB-22-8022, Neobiotech), and were used at a 1:200 dilution. Retrieval was performed with citrate buffer (pH 6.0) using a pressure cooker. Reactions were visualized using the EnVision anti-mouse antibody system, and developed using the DAB-Plus Kit (Dako, Copenhagen, Denmark). Slides were counterstained with Harry's modified Hematoxylin.

### Microsatellite genotyping

We compared the amplification pattern of D5S299, D5S346, D3S1612, D5S82 and D3S3564 genomic microsatellites between the generated tumor xenograft and the patient's skin biopsy from which it was derived. DNA was extracted from a fresh fragment of the tumor xenograft (passage 1) using the phenol/chloroform/isoamyl method, and from five slides of ten mycras of a formalin-fixed fragment of the patient's skin lesion using a DNA QiAamp FFPE tissue kit (Qiagen), following the manufacturer's instructions. First, the microsatellites were amplified by PCR using labeled primers. The primer sequences were as follows: D3S1612 UP-5′-(YAKYE)GCTCTCCTCAGTGGAAAATT-3′ and DW-5′-ATGTAGAAGAGGATGATCTCC-3′; D3S3564 UP-5′-(FAM)AGCTAAACACAGTCTAACTGCAT-3′ and DW 5′-CCCACAGAGTGATAGGGA-3′; D5S299 UP-5′-(ATTO550)GTAAGCAGGACAA GATGACAG-3′ and DW 5′-GCTATTCTCTCAGGATCTTG-3′; D5S82 UP-5′-(FAM)CCCAATTGTATAGATTTTAGAAGTC-3′ and DW 5′-ATCAGAGTATCAGAATTTCT-3′; and D5S346 UP-5′-(FAM)ACTCACTCTAGTGATAAATCGGG-3′ and DW 5′-AGCAGATAAGACAGTATTACTAGTT-3′. All microsatellites were amplified in the same conditions but in separated reactions, starting with 50 ng of DNA and with 5 µl of Mega Mix double (Microzone) and 10 µM of each labeled primer in a final volume of 10 µl. Amplifications were carried out using an initial denaturing step at 96°C for 5 min, 35 cycles of denaturing at 96°C, annealing at 55°C (D5S299/ D5S82/ D5S346) or 58° (D3S1612/D3S3564), and extension steps at 72°C (30 s each), and a final extension step of 72°C for 3 min. The PCR products were first visualized in a 1.5% agarose gel and then diluted 1/100 in a buffer containing HI-DI formamide and Genescan 500 LIZ standard marker for their visualization and analysis in a capillary sequencer (ABIPRISM 3130XL, Applied Biosystems) using GeneScan Analysis (Thermo Fisher Scientific).

### Analysis of genomic imbalances

Cytogenetic analysis was performed with DNA from tumor xenograft (passage 1) using a CytoScan 750K Array (Affymetrix, Thermo Fisher Scientific) with genome-wide coverage (750,000). The sample was processed in the GeneChip System 3000 platform from Affymetrix (Thermo Fisher Scientific). The Chromosome Analysis Suite program was used for the analysis (v.3.2, Thermo Fisher Scientific) with version 33.1 that uses the UCSC hg19 version of the human genome for the annotations of genes. The alterations were analyzed with a minimum of 25 affected markers and an average resolution of 110 kb.

### Karyotype and FISH analysis

PDOX tumor cells were dissociated using collagenase, and then cultured in RPMI medium (GIBCO, Thermo Fisher Scientific) with 20% fetal bovine serum (FBS; GIBCO, Thermo Fisher Scientific), insulin (2.5 UI/ml) and 1× streptomycin and penicillin (GIBCO, Thermo Fisher Scientific) for 4 days. Conventional cytogenetic analysis was performed on cultured cells and chromosome G-banding was carried out using standard techniques. Interphase FISH was performed on fixed cells from bone marrow at diagnosis and from dissociated and cultured tumor cells with the following probes: XL IGH Break Apart Probe; XL t(4;14) FGFR3/IGH Translocation/Dual Fusion Probe; and XL t(14;16) IGH/MAF Translocation/Dual Fusion Probe (MetaSystems).

### Whole-exome sequencing

Genomic DNA from the xenograft tumor was fragmented to an average size of 150 bp and subjected to DNA library creation using established Illumina paired-end protocols. Adapter-ligated libraries were amplified and indexed via PCR. The Agilent SureSelect All Exon V6 library preparation kit (without UTR, Agilent) was used and sequence targets were captured and amplified in accordance with the manufacturer's recommendations. Enriched libraries were subjected to 150 base paired-end sequencing (Illumina Novaseq System) following the manufacturer's instructions. Quality control of raw reads was performed using FastQC (version 0.11.7) (Wingett and Andrews, 2018). Raw reads were trimmed using Timmomatic software (version 0.38) (Bolger et al., 2014). Trimmed reads were aligned to the human genome (version hg38 from the UCSC) using the BWA-MEM algorithm (www.arxiv.org/abs/1303.3997) from BWA (version 0.7.17) (Li and Durbin, 2010) with default parameters. Resulting BAM files were sorted and indexed using Sambamba (version 0.6.8) (Tarasov et al., 2015). Duplicate reads were identified and removed from sorted BAM files using Picard Tools (version 2.18.16) (http://broadinstitute.github.io/picard). For the variant calling, filtering and annotation, SNVs and short indels were called with the Genome Analysis Toolkit (GATK) (version 3.8-1-0) with default parameters (McKenna et al., 2010). SNVs and indels were separately filtered using the recommended variant quality score recalibration according to GATK best practice recommendations (Depristo et al., 2011). Filtered called variants were annotated with dbSNP 146 rsIDs, the 1000 Genomes Phase 1 high-confidence SPNs release, and the Mills and 1000G gold standard indels. Prediction of effects was performed using snpEff (Cingolani et al., 2012).

### Methylation analysis

Genome-wide DNA methylation analysis of EMM xenograft tumor was performed using an Infinium Methylation EPIC BeadChip kit (Illumina), following the manufacturer's recommendations. Four different tumor fragments were included (passage 1) to cover possible tumor heterogeneity. DNA was extracted using a phenol/chloroform/isoamyl protocol. The quality of the DNA was checked by electrophoresis using a 1% agarose and 1/100,000 SyberSafe gel, and quantified using a Nanodrop-1000 spectrophotometer. For bisulfite conversion, we started with 600 ng of each sample, and a EZ DNA methylation kit (Zymo Research) was used according to the manufacturer's recommendations for the Illumina Infinium assays. The effectiveness of the bisulfite conversion was checked for three controls that were converted simultaneously with the samples. Bisulfite-converted DNA (4 μl) was used for hybridization on Infinium Methylation Epic BeadChip, following the Illumina Infinium HD Methylation protocol. The array was evaluated using the Illumina HiScan SQ scanner. The intensities of the images were extracted using the minfi 1.30 R package and normalized using the ssNoob method. The Illumina control probes and the snp-containing probes were removed from subsequent analysis. The methylation score for each CpG was represented as a beta value according to the fluorescent intensity ratio. Beta values may take any value between 0 (non-methylated) and 1 (completely methylated). Differential methylation analysis was performed using the MEAL v1.14 R library and CpGs with an absolute beta value difference greater than 0.2 and a false discovery rate lower than 0.05 were selected. For comparison, a published DNA methylome dataset of 101 newly diagnosed and chemotherapy-naïve MM patients and NPCs obtained using HumanMethylation450 Beadchip (Illumina) were included (Agirre et al., 2015). In that dataset, NPCs were composed from three normal bone marrows (pools from four patients each) and eight non-tumoral tonsils. In order to compare the results with the published 450K methylation microarrays, only the common probes with EPIC were taken into account, and average methylation values were used for each group. The selected CpGs were annotated with the UCSC hg19 genome version and the categories TSS 1500, TSS 200, 5′ UTR, first exon, gene body, 3′ UTR and intergenic, related to their gene location.

### *In vivo* therapeutic assays

For *in vivo* therapeutic assays, tumor was expanded by orthotopic implantation in 40 additional 6-week-old female NSG mice, and when tumor volumes raised 400-500 mm^3^ homogeneous size, animals were distributed randomly to four experimental groups (*n*=10) and treated for 15 days with: (1) group 1 – control (saline intraperitoneally five times a week); (2) group 2 – daratumumab (three doses of 20 mg/kg intraperitoneally on day 1, 7 and 13); (3) group 3 – lenalidomide (1 mg/kg, oral) plus dexamethasone (15 mg/kg, intraperitoneally), four consecutive doses of both with an interval of 2 days between each cycle (total of twelve doses); and (4) group 4 – carfilzomib (6 mg/kg, i.p., two consecutive doses at days 1-2, 6-7 and 12-13) plus lenalidomide plus dexamethasone (same regime as described in group 3). During treatment, tumor growth and animal weight were monitored twice a week. Tumor volume was estimated by measuring tumor width (*W*, mm) and length (*L*, mm) with a caliper, and using the formula v=(π×L×W^2^)/6. At the end of treatment, animals were sacrificed and tumors were harvested, weighed and processed for histological, immunohistochemical and molecular studies.

### Western blot analysis

Protein extraction was performed using RIPA buffer [50 mM Tris-HCl (pH 7.4), 150 mM sodium chloride, 1 mM ethylenediaminetetraacetic acid, 1% NP-40, 1% sodium deoxycholic acid and 0.1% sodium dodecylsulfate] with the addition of protease and phosphatase inhibitor cocktails (Roche). Cyclin D (sc-8396), Cyclin E (sc-247), Bcl-X_L_ (sc-8392) and tubulin (sc-5286) antibodies were purchased from Santa Cruz Biotechnology and were used at a 1:1000 dilution. p-FAK (8556, used at a 1:500 dilution), p53 (2524, used at a 1:2000 dilution), AKT (4691, used at a 1:1000 dilution) and P-elf2alfa (3398, used at a 1:1000 dilution) antibodies were obtained from Cell Signaling Technology. The CD38 (NB-22-6755) antibody was purchased from Neobiotech and was used at a 1:1000 dilution. The TUNEL assay (ApopTag Peroxidase *In Situ* Apoptosis kit, Millipore) was performed following the manufacturer's recommendations.

### Derivation of EMM cell line

To derive the EMM cell line, a xenograft tumor fragment of 125 mm^3^ from passage 2 was first excised manually using two sterile scalpels with small fragments in a sterile 100 mm culture dish with 8 ml of Dulbecco's modified Eagle medium (DMEM) medium containing 15.000 IU of IV collagenase. The suspension of fragments was then transferred to a 50 ml conical centrifugation tube and digested in the collagenase solution for 1 h at 37°C. During this period, the tube was swirled vigorously every 15 min. The cell suspension was then disaggregated by pipetting, filtered through a 100 μm cell strainer (Corning, Merck) and centrifugated at 180 ***g*** for 10 min. The cell pellet was rinsed with PBS and counted in a chamber slide using an automated cell counter (Countess II, Thermo Fisher Scientific). Cells were re-centrifuged in the same conditions and resuspended in DMEM containing 5% glutamine (DMEM Glutamax, GIBCO, Thermo Fisher Scientific) and complemented with 20% FBS, 1% streptomycin and penicillin, 10 μg/ml of insulin and 30 μg/ml of transferrin (all obtained from Thermo Fisher Scientific) to a concentration of 2.5×10^4^ cell/ml in a T-25 flask. Cells were cultured at 37°C in a CO_2_ incubator. To perform different passes, cells were brought into a single-cell suspension by pipetting. Then cells were counted in a chamber slide using an automated cell counter. Then, cells were rinsed with PBS (Thermo Fisher Scientific) by centrifugation at 180 ***g*** for 10 min and re-suspended in a 50:50 mixture of new and reconstituted medium (from the previous passage), and subcultured by dilution. Cells were cryopreserved in FBS with 10% DMSO. All protocols were performed using sterile techniques in a Class II Type A2 laminar flow hood. To test whether the EMM-derived cell line could generate new plasmocytomas, 1×10^6^ cells soaked in 50 µl of Matrigel (BD Biosciences) were intradermally injected in two flanks of three NSG mice (total of 6 injections). Tumor growth was monitored every 5 days post-injection by measuring tumor width (*W*, mm) and length (*L*, mm) with a caliper until day 30 post-injection when mice were sacrificed. Tumor volume was estimated using the same formula indicated above. At sacrifice, tumors were harvested and processed for histological and IHQ studies.

### Statistical analysis

Statistical analysis was performed in WES, and methylation studies were described in the corresponding sections. Tumor weights were expressed in mg and tumor volumes in mm^3^, and represented as mean±s.e.m. Tumor volumes, as well as final tumor weight, were compared between each treatment group and control group using a parametric Student's *t*-test and GraphPad Prism v7 Software.

## Supplementary Material

Supplementary information
