# Supplementary material for: Extramedullary multiple myeloma patient-derived orthotopic xenograft with a highly altered genome: combined molecular and therapeutic studies
Source: Dis Model Mech. 2021 Jul 15;14(7):dmm048223. doi: 10.1242/dmm.048223 (PMC8325009; doi:10.1242/dmm.048223)
Supplement: Supplementary information [file dmm-14-048223-s1.pdf]

FIGURE S1

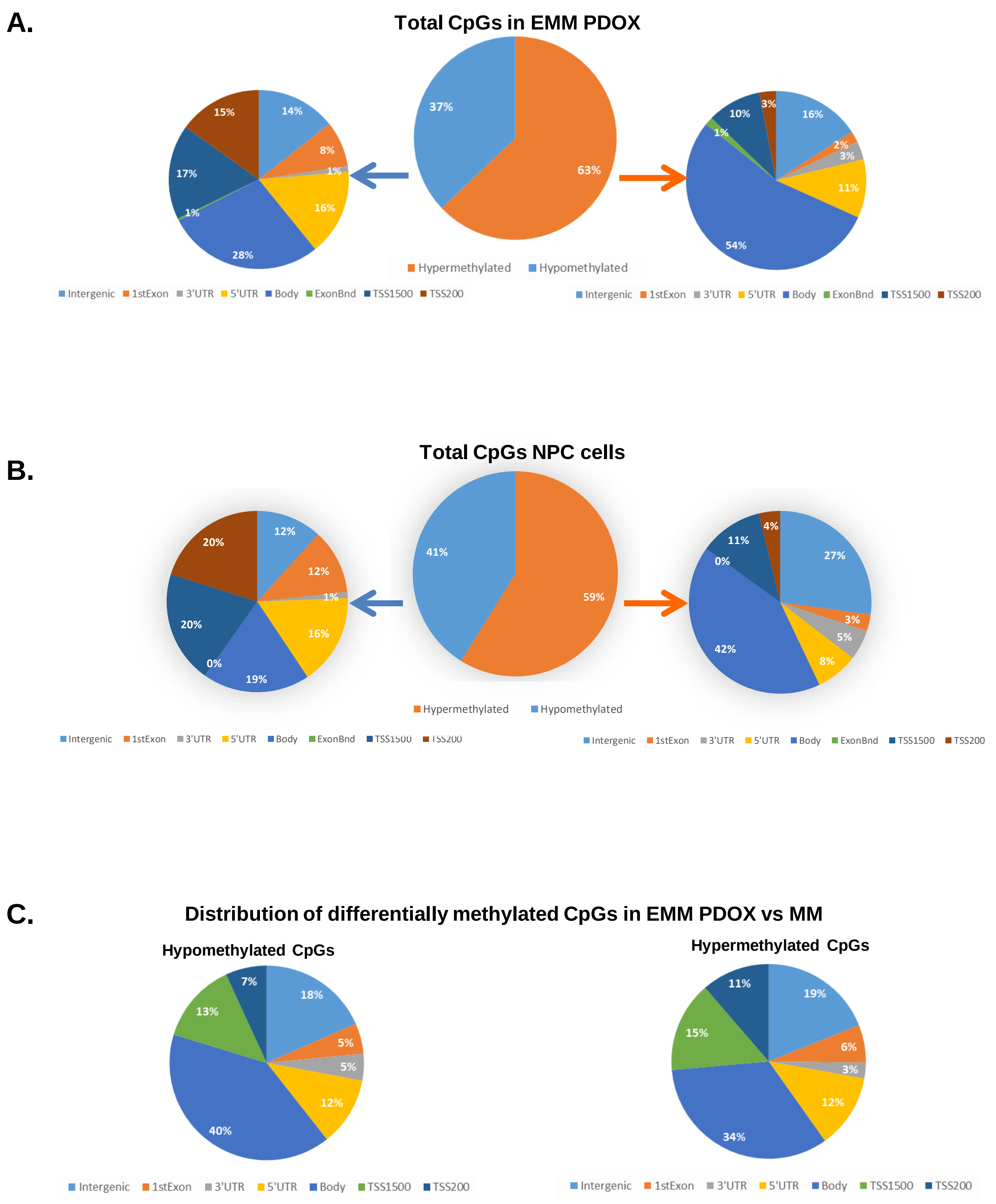

Disease Models & Mechanisms • Supplementary information

Figure S1. Distribution of hypermethylated and hypomethylated CpGs by genome functional regions. **(A)** Percentage of total hypermethylated and hypomethylated CpGs in EMM PDOX and their distribution by genome functional regions (hypermetylated CpGs (left panel) and hypomethylated CpGs (right panel)). **(B)** Percentage of total hypermethylated and hypomethylated CpGs in NPC and their distribution by genome functional regions (hypermetylated CpGs (left panel) and hypomethylated CpGs (right panel)). **(C)** Distribution of differentially methylated CpGs between EMM PDOX and 101 newly diagnosed MM cases (hypermethylated CpGs (left panel) and hypomethylated CpGs (right panel)). NPC and MM cases and data were previously reported in Agirre et al., 2015.

Table S1

Click here to download Table S1

Table S2

Click here to download Table S2

Table S3

Click here to download Table S3
